# Identifying the Transcriptional Regulatory Network Associated With Extrathyroidal Extension in Papillary Thyroid Carcinoma by Comprehensive Bioinformatics Analysis

**DOI:** 10.3389/fgene.2020.00453

**Published:** 2020-05-11

**Authors:** Yong Chen, Bo Jiang, Wenlong Wang, Duntao Su, Fada Xia, Xinying Li

**Affiliations:** Department of General Surgery, Xiangya Hospital, Central South University, Changsha, China

**Keywords:** papillary thyroid carcinoma, extrathyroidal extension, weighted gene co-expression network analysis, transcription factor, long noncoding RNA

## Abstract

Extrathyroidal extension (ETE) affects papillary thyroid cancer (PTC) prognosis. The objective of this study was to identify biomarkers for ETE and explore the mechanisms controlling its development in PTC. We performed a comprehensive bioinformatics analysis using several datasets. Differential expression analysis and weighted gene co-expression network analysis (WGCNA) on 58 paired PTC samples from The Cancer Genome Atlas (TCGA) were used to detect ETE-related mRNA and long noncoding (lnc) RNA modules and construct an lncRNA/mRNA network. An independent TCGA dataset containing 438 samples was utilized to validate and characterize the WGCNA results. Functional annotation was used to identify the biological functions and related pathways of ETE modules. Two independent RNA sequencing datasets were combined to crossvalidate relationships between lncRNAs and mRNAs by Pearson correlation analysis. Transcription factors (TFs) for affected genes were predicted using the binding motif data from Ensembl Biomart to construct a TF/lncRNA/mRNA network. Other two independent datasets were used to crossvalidate TF-mRNA associations. Finally, receiver operating characteristic, survival analyses, and Cox proportional hazard regression model were performed to explore the significance of hub genes in ETE diagnosis and PTC prognosis. Three mRNA modules and two lncRNA modules were significantly associated with ETE. Enrichment analysis showed extracellular matrix changes was closely related to the development of ETE. A TF/lncRNA/mRNA regulatory network was constructed containing 33 validated hub genes, 64 lncRNAs, and 64 TFs, all differentially expressed between ETE and non-ETE samples. Unc-5 family C-terminal like [area under the curve (AUC): 0.711], sushi repeat containing protein X-linked 2 (AUC: 0.706), lysyl oxidase (AUC: 0.704), collagen type I alpha 1 chain (AUC: 0.704), and collagen type X alpha 1 chain (AUC: 0.704) were the most highly significant hub genes for ETE diagnosis. The Cox proportional hazard regression model constructed with hub genes showed significant survival differences between low- and high-risk groups (*p* = 0.00025) and performed good prediction for PTC prognosis(AUC = 0.794; C-index = 0.895). The identification of 33 biomarkers and TF/lncRNA/mRNA regulatory network would provide new insights into the molecular mechanisms of ETE besides the prognosis model may have important clinical implications in the improvement of PTC risk stratification, therapeutic decision-making, and prognosis prediction.

## Introduction

Papillary thyroid cancer (PTC) is the most common type of thyroid cancer (TC), accounting for approximately 90% of all cases (Kitahara et al., 2017; Xia et al., 2019). It has relatively good prognosis and a low mortality rate (Xia et al., 2018). However, its prognosis is associated with several clinicopathological factors, including age, tumor size, distant metastasis, and the development of extrathyroidal extension (ETE; Hay et al., 1993; So et al., 2015; Lee et al., 2017).

Extrathyroidal extension, defined as the invasion of the primary tumor into adjacent tissues beyond the thyroid gland, is an important prognostic factor (McConahey et al., 1986; Cady and Rossi, 1988). Woolner et al. (1961) first reported the adverse effects of ETE on the prognosis of patients with PTC. Since then, many studies have been reported that patients with PTC who were discovered to have maximal ETE during surgery have an increased risk of tumor recurrence and death, and this idea is now widely accepted (McConahey et al., 1986; Hay et al., 2002). According to the current eighth edition of the American Joint Committee on Cancer staging system, ETE is an important factor in the prognostic staging of differentiated TC. Based on the scope of ETE, PTC is considered stage II if only the strap muscles are grossly invaded (T3b) and stage III with gross invasion of the subcutaneous tissue, larynx, trachea, esophagus, or recurrent laryngeal nerve (T4a; Perrier et al., 2018). This means that ETE plays an important role in PTC risk stratification, treatment strategy decisions, and survival prognosis. However, despite its significance, few reports exist on the molecular mechanisms controlling ETE.

Rapid advancements in biological technology and bioinformatics have resulted in new more effective methods to study the mechanisms of cancer. In more than 30 years, sequencing technology has made considerable progress, it is becoming more and more effective and accurate. Expression profiles such as microarray and RNA-sequencing have been widely used to identify the gene expression level. Chromatin immunoprecipitation (ChIP) is a powerful tool for studying protein-DNA interactions *in vivo* and is often used to study transcription factor binding sites or histone-specific modification sites. Besides, many other biotechnologies have been invented, such as single-cell sequencing, methylation sequencing, etc. (Furey, 2012; Thermes, 2014; Stark et al., 2019). While these technologies bring large amounts of biology data for research, they also bring huge challenges for biostatistics. How to effectively use these data, the rapid development of bioinformatics is crucial. It can help us mine more information in the data. Weighted gene co-expression network analysis (WGCNA) is an effective bioinformatics method that characterizes correlation patterns among genes in microarray or RNA sequencing (RNA-seq) samples. It can be used to identify modules with highly correlated genes, to relate modules to external sample traits, and to calculate module membership (MM) measures. More importantly, correlation network-based gene screening methods can be used to identify candidate biomarkers or treatment targets (Langfelder and Horvath, 2008). This method has been successfully applied to a variety of diseases (Chen et al., 2018; Zhang et al., 2018; McDonough et al., 2019). The Cox proportional-hazards regression model has achieved widespread use in the analysis of time-to-event data with censoring and covariates. The exponential of the coefficients from the Cox model gives the instantaneous relative risk for an increase of one unit for the covariate in question. It is often used to predict survival in terms of subject covariates (Fisher and Lin, 1999). The purpose of this study was to explore the molecular mechanisms of ETE development in PTC and identify ETE biomarkers through comprehensive bioinformatics analysis. The results provide new insights to the genes regulating ETE.

## Materials and Methods

### Data Collection

An RNA-seq dataset containing 568 TC samples was downloaded from The Cancer Genome Atlas (TCGA) database^1^ for differentially expressed gene (DEG) screening, WGCNA, and validation. Matched clinical data were obtained from University of California Santa Cruz (UCSC) Xena^2^. Datasets GSE83520 and GSE64912, derived using Illumina HiSeq RNA-seq technology and obtained from the Gene Expression Omnibus (GEO) database^3^, were combined and analyzed to crossvalidate relationships between long noncoding (lnc) RNAs and mRNAs. The human reference genome (version: GRCh38.p12) and related human binding motif data were downloaded from the Ensemble BioMart database^4^ to predict transcription factors (TFs) affecting identified genes. A gene expression matrix [log2 (fragments per kilobase of transcript per million mapped reads)] of 568 TC samples was obtained from UCSC Xena to correlate TFs with lncRNAs and mRNAs. The microarray datasets GSE33630 and GSE60542, both acquired using the Affymetrix Human Genome U133 Plus 2.0 Array (HG U133 Plus 2.0) platform, were obtained from the GEO database, combined, and analyzed to crossvalidate relationships between TFs and mRNAs.

### Data Preprocessing and Differentially Expressed Gene Screening

We preprocessed the raw data from microarray and RNA-seq experiments in different ways. For RNA-seq data, the read counts data were transformed by variance-stabilizing transformation in the R package “DESeq2” (Chen et al., 2018). For microarray data, raw expression data were calculated following preprocessing, including robust multi-array analysis background correction, log2 transformation, and quantile normalization. Probes were annotated using Affymetrix annotation files, and when different probes were linked to the same gene, the average value was used as the gene expression value. As different datasets and different samples within datasets were processed in multiple batches, batch effects were corrected using the ComBat method implemented in the “SVA” package. After data preprocessing, principal component (PC) analysis was used to verify dataset quality using the R package “FactoMineR” (Supplementary Figure S1). “DEseq2” was applied to identify DEGs in 58 pairs of PTC samples from TCGA. The cut-off criterion for DEGs was an adjusted *p* value (adj*P*) <0.05, and these DEGs, including lncRNAs and mRNAs, were used for further WGCNA analysis.

### Weighted Gene Co-expression Network Construction

The R package “WGCNA” was used to construct a co-expression network based on the gene expression profiles of differentially expressed mRNAs and lncRNAs from the 58 PTC samples with complete clinical data. The process of constructing the mRNA co-expression network was the same as for lncRNA. First, by calculating the correlations between all pairs of genes, a matrix of similarity was constructed. Then, the integrated function pickSoftThreshold in the “WGCNA” package was used to select an appropriate soft-thresholding power β. With this soft-thresholding power, the matrix of similarity was raised to result in scale-free topology. Third, the adjacency matrix was transformed into a topological overlap matrix by similarity, and the corresponding dissimilarity was also calculated. Finally, co-expression gene modules were identified using the R package “Dynamic Tree Cut” with a deepSplit of 2, a minModuleSize of 30, and a maxBlockSize of 20,000. Module eigengenes (i.e., the first PC of the gene expression matrix in each module) were obtained by WGCNA and represented the expression profiles of their corresponding module genes. Highly similar modules were merged when the module eigengene height in the clustering was <0.25.

### Identifying Clinically Significant Modules and Module Functional Annotation

Weighted gene co-expression network analysis identifies gene modules based on their expression similarities in samples, and calculates correlations between external clinical information and gene modules to identify clinically significant modules. Gene modules most correlated with ETE were selected as ETE-related modules, and the functions of the genes these modules were explored through gene ontology (GO) enrichment analysis and Kyoto Encyclopedia of Genes and Genomes (KEGG) pathway enrichment analysis, performed using the R package “clusterProfiler,” and using adj*P* < 0.05 as a threshold for inclusion.

### Hub Gene Identification and Validation

Genes that had the highest degrees of connectivity in their gene modules and helped determine the module characteristics were termed hub genes, and were identified by calculating gene significance (GS) and MM. The GS of a gene is the correlation between the gene and a relevant clinical parameter, and the MM of a gene is the correlation between its expression profile and those of identified module eigengenes. Genes with absolute values of MM > 0.85 and GS > 0.4 and *p* values <0.05 for both MM and GS were defined as hub genes in their modules. An independent dataset of 438 PTC samples from TCGA with complete clinical data were analyzed to compare the expression of hub genes between ETE and non-ETE samples using the Wilcoxon rank sum test.

### lncRNA/mRNA Network Construction

Clinically relevant lncRNA modules were identified in the same manner as for mRNAs, but the criteria for lncRNAs were *p* values <0.05 for both GS and MM. The 58 pairs of PTC samples from TCGA were used in correlation analysis to construct the lncRNA/mRNA network, and two independent RNA-seq datasets (GSE64912 and GSE83520) were combined to crossvalidate the results. The criteria for screening the regulatory lncRNAs of hub mRNAs was an absolute correlation coefficient (*r*) value >0.6 and a *p* value <0.05. In addition, the independent dataset of 438 PTC samples was analyzed to compare the expression levels of these regulatory lncRNAs between patients with and without ETE.

### TF/lncRNA and TF/mRNA Network Construction

The Human Reference Genome data and human binding motif data (both version GRCh38.p12) were downloaded from Ensembl BioMart. The promoter region of a gene was defined as the 1,000 bp upstream and 200 bp downstream of the transcriptional start site, and the TFs binding the promoters of regulatory lncRNAs and hub mRNAs were predicted according to known TF binding motifs. The specific process for our team to predict the transcription factor of the target genes was shown in Supplementary Table S1.

Then, the gene expression matrix from UCSC Xena was used in Pearson correlation analysis to associate TFs with lncRNAs and mRNAs. In addition, the microarray datasets GSE33630 and GSE64512 were combined and analyzed to crossvalidate relationships between TFs and mRNAs. The criterion for assigning TFs to regulatory lncRNAs and hub mRNAs was a *p* value <0.05. In addition, associations between identified TFs and ETE were validated with a dataset of 438 PTC samples using the Wilcoxon rank sum test.

### TF/lncRNA/mRNA Network Construction

Through the above series of analyses, we obtained hub genes associated with ETE, and regulatory lncRNAs significantly co-expressed with hub mRNAs. These were combined with the predicted TFs of hub mRNAs and regulatory lncRNAs to construct a complete TF/lncRNA/mRNA regulatory network for PTC-related ETE in Cytoscape (version: 3.7.1).

### Receiver Operating Characteristic and Survival Analyses

To detect the significance of hub genes for the diagnosis of ETE in PTC, receiver operating characteristic (ROC) analysis was performed with an independent subset with 438 PTC samples and the area under the curve (AUC) was calculated using the R package “plotROC” (version: 2.2.1). An AUC > 0.5 indicated upregulation, while an AUC < 0.5 represented downregulation. Larger | AUC-0.5| values indicated that these genes had increased ability to distinguish between ETE and non-ETE samples.

To reveal the prognostic value of hub genes in patients with PTC, survival analysis was performed using the GEPIA database^5^. Overall survival (OS) calculations and statistical significance assessments were performed using the Kaplan-Meier (KM) method (Paszek et al., 2005) and the log-rank test (Butcher et al., 2009), respectively. *p* values <0.05 were considered statistically significant. To further clarify how hub genes collectively affect patient survival and prognosis, we first performed a one-way ANOVA analysis on clinical characteristics of 501 PTC from TCGA to determine the reliability of the clinical data (Supplementary Table S9). Then we constructed Cox proportional hazard regression model using hub genes and survival information, and Akaike information criterion (AIC) was used to optimize the cox regression model which is a measure of the goodness of fit of a statistical model by the R packages Survival and Stats (Terry et al., 2000). Based on the median of the risk scores, we classified all 501 PTC patients into high- and low-risk two groups. Then KM curves and log-rank test were used to assess survival differences between the two groups. Besides, two different methods including time-dependent ROC curves and concordance index (C-index) were used to measure the accuracy of the Cox regression model, and the stats of the two methods were compared using bootstrap with 1,000 times re-sampling separately to ensure the credibility (Heagerty et al., 2000; Schröder et al., 2011). Finally, the nomogram was developed based on the result of the multivariable Cox regression model to detect the effect of each hub genes on patient survival prognosis. A weighted score calculated using hub genes was used to estimate 3- and 5-year OS. All statistical analysis was performed by R version 3.6.3 statistical software.

## Results

### Data Preprocessing

**FIGURE 1:**
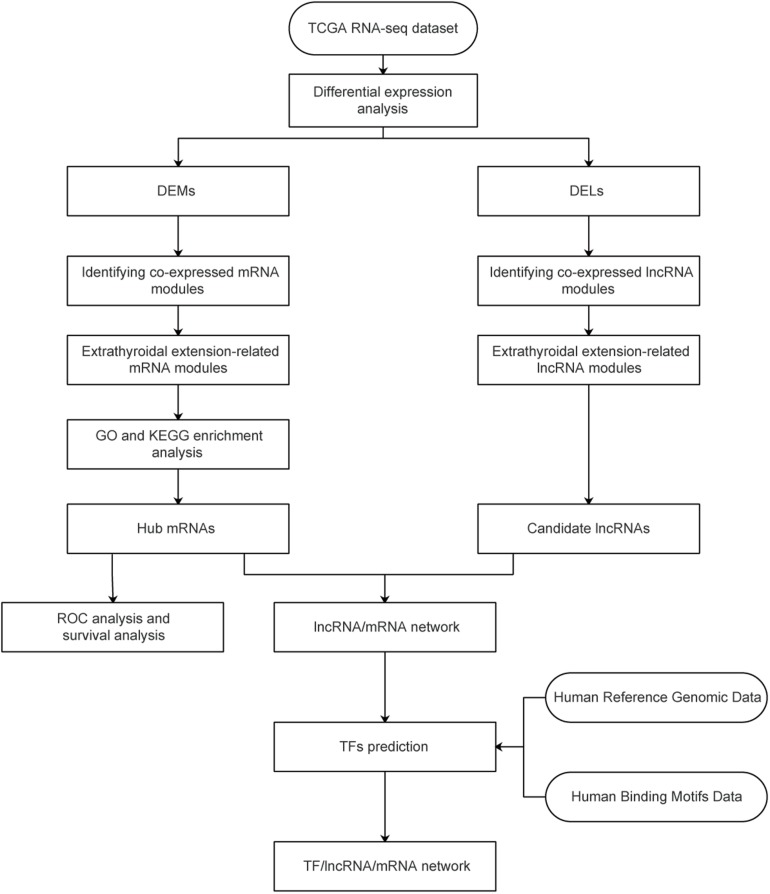
Study flowchart. TCGA, The Cancer Genome Atlas; GO, gene ontology; DEG, differentially expressed gene; DEL, differentially expressed lncRNA.

A workflow of the study is shown in Figure 1. A level three RNA-seq dataset containing 510 thyroid tumor samples and 58 normal samples was obtained from the TCGA database. After removing one follicular TC sample, eight metastatic samples, and five samples without complete ETE clinical information, 496 primary tumor samples, and 58 healthy samples from patients with PTC remained. These 554 samples were divided into two subsets: one, called TCGA58, contained tumor samples and matched healthy samples from 58 patients with PTC; the other, called TCGA438, contained tumor samples from the remaining 438 patients. Details on the datasets used in this study are presented in Supplementary Table S2.

### Differentially Expressed Gene Identification

**FIGURE 2:**
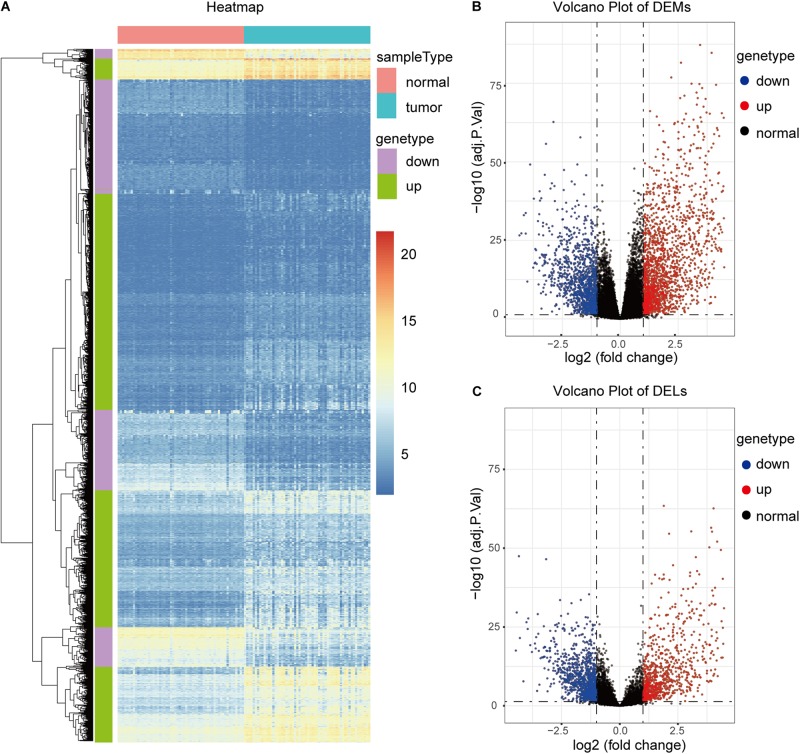
Identification of DEGs in the TCGA58 dataset. **(A)** Heatmap of the top 2,000 DEGs. **(B,C)** Volcano plots of DEMs and DELs.

TCGA58 was subjected to differential expression analysis, and 16,134 DEGs, including 11,584 mRNAs and 4,550 lncRNAs, were identified. With a | log2 (FC)| > 1 and adj*P* < 0.05, 4,991 significant DEGs, including 3,095 mRNAs and 1,896 lncRNAs, were identified. Among the significant DEGs, 2,699 and 2,292 were significantly upregulated and downregulated in the cancer samples, respectively (Figures 2A–C).

### Weighted Co-expression Network Construction

**FIGURE 3:**
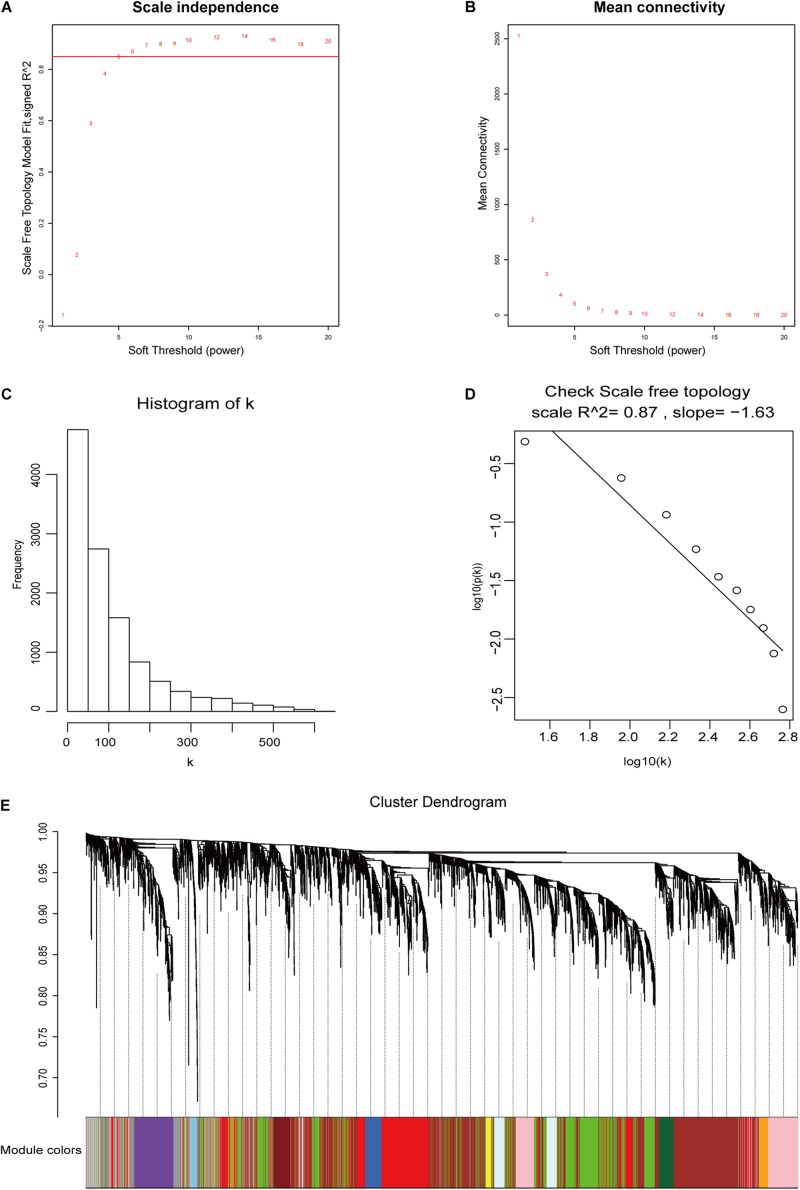
DEG weighted gene co-expression network construction. **(A,B)** The scale-free fit index and the mean connectivity with various soft-thresholding powers. **(C,D)** Histogram of connectivity distributions and check scale-free topology when β = 5. **(E)** DEG clustering dendrograms.

The 11,584 differentially expressed mRNAs, 4,550 differentially expressed lncRNAs, and 58 tumor samples with complete clinical data from TCGA58 were used to construct mRNA and lncRNA weighted co-expressed networks. One of the most critical parameters was the soft-thresholding power value, which affected the independence and average connectivity degree of co-expression modules. The appropriate soft-thresholding power was selected when the scale-free topology fit index reached 0.85. In the mRNA weighted co-expressed network, a power value of five was selected, which identified 30 modules (Figures 3A–E). In the lncRNA weighted co-expressed network, a power value of six was selected, which identified 15 modules (Supplementary Figure S2).

### Identification of Clinically Significant Modules and Module Functional Annotation

**FIGURE 4:**
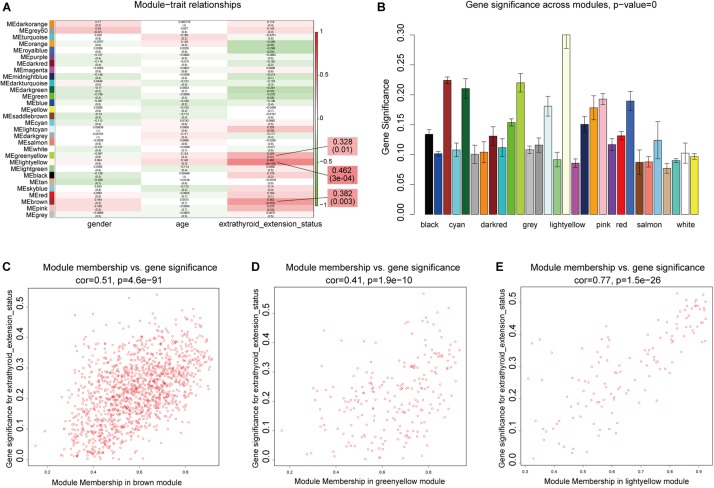
Identification of mRNA modules associated with clinical traits of ETE. **(A)** Heatmap of module–trait relationships. **(B)** Distribution of average gene significance in modules related with ETE. **(C–E)** Scatter plots of correlations between gene module membership and gene significance in the three ETE-related mRNA modules.

We next sought to identify modules most significantly related to clinical features, and investigate their biological functions. In the mRNA network, based on threshold criteria of | *r*| > 0.3 and *p* < 0.05, brown, greenyellow, and lightyellow modules were defined as most relevant to ETE (Figures 4A–E). These three modules contained 1,362, 223, and 129 mRNAs, respectively. In the lncRNA network, with the same criteria, salmon and turquoise modules were defined as the most relevant to ETE (Supplementary Figure S3); these two modules contained 65 and 653 lncRNAs, respectively.

To examine the functions of the three ETE-related mRNA modules, genes in these modules were subjected to GO enrichment analysis, and the detailed results are shown in Supplementary Table S3. Interestingly, in cellular component analysis, all three modules were enriched for genes of the extracellular matrix (ECM), which is a major component of the tumor microenvironment and plays important roles in tumor progression. In terms of biological processes, the lightyellow and greenyellow modules were both enriched in genes involved in ECM regulation, including the terms “extracellular matrix organization” and “extracellular structure organization,” while the brown module was enriched in genes involved in the regulation of the immune response, which is also closely associated with tumorigenesis, invasion and metastasis. Enriched terms included “granulocyte chemotaxis,” “neutrophil chemotaxis,” and “leukocyte chemotaxis.”

Detailed results from the KEGG analysis are shown in Supplementary Table S4. The lightyellow module was mainly enriched in pathways controlling ECM remodeling, including protein digestion and absorption, ECM-receptor interactions, focal adhesions, and the phosphoinositide 3-kinase/Akt signaling pathway, while the brown module was mainly enriched in pathways controlling the immune response, such as cytokine–cytokine receptor interactions, interleukin 17 signaling, and chemokine signaling. The greenyellow module was enriched for signal transduction pathways, including neuroactive ligand–receptor interactions, transforming growth factor β signaling, and phospholipase D signaling, all of which are associated with tumor progression.

### Identification and Validation of Hub Genes

**FIGURE 5:**
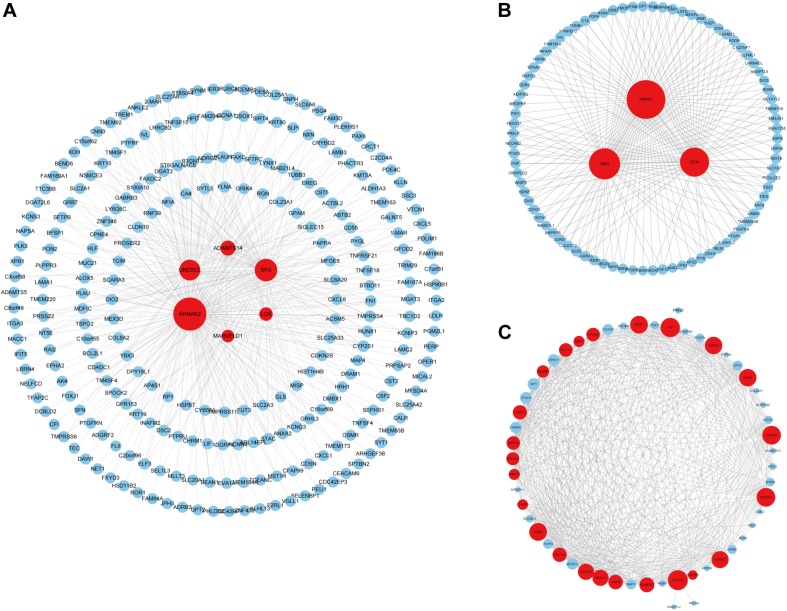
Construction of hub gene-centric interaction networks. **(A)** An interaction network centered on six hub genes in the brown module. **(B)** An interaction network centered on three hub genes in the greenyellow module. **(C)** An interaction network centered on 24 hub genes in the lightyellow module. Hub genes are shown in red and the point size indicates the node degree.

**FIGURE 6:**
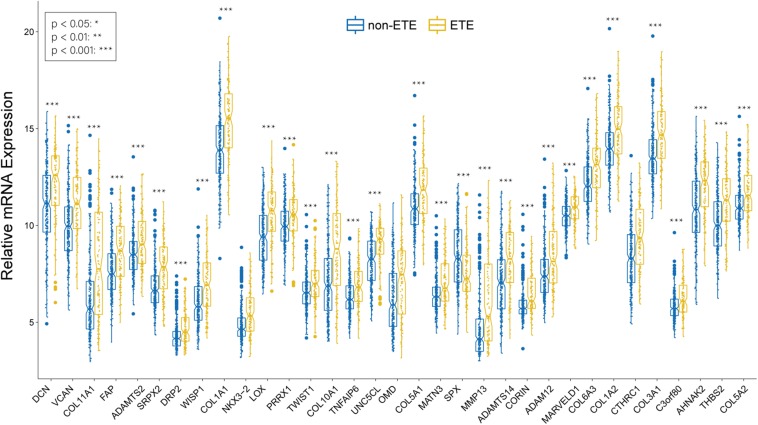
Boxplots of hub genes between ETE and non-ETE samples in the TCGA438 dataset. *p* values were calculated using the Wilcoxon rank sum test. *p < 0.05, **p < 0.01, and ***p < 0.001.

We identified 33 highly connected genes in the brown, greenyellow, and lightyellow modules as hub genes (Table 1), and generated hub gene-centric networks (Figures 5A–C). The hub genes were validated using the TCGA438 dataset, and significant expression differences in ETE and non-ETE samples were detected for each hub gene (Figure 6).

**TABLE 1:** Hub genes in modules associated with ETE.

Gene symbol	Module	GS	MM	GS *p*-value	MM *p*-value
AHNAK2	Brown	0.402278	0.873431	0.001746	3.81E−19
ADAMTS14	Brown	0.43586	0.868652	0.000626	1.01E−18
MARVELD1	Brown	0.43485	0.863324	0.000647	2.85E−18
LOX	Brown	0.462729	0.863266	0.000255	2.88E−18
UNC5CL	Brown	0.405672	0.860998	0.001582	4.42E−18
SPX	Brown	−0.44091	−0.86006	0.000532	5.27E−18
PRRX1	Greenyellow	0.403475	0.915264	0.001686	8.94E−24
OMD	Greenyellow	0.518178	0.88662	3.10E−05	2.10E−20
DCN	Greenyellow	0.418423	0.855051	0.001081	1.32E−17
ADAM12	Lightyellow	0.447943	0.922858	0.000422	7.15E−25
COL6A3	Lightyellow	0.501222	0.92266	6.13E−05	7.66E−25
COL5A1	Lightyellow	0.474416	0.918535	0.000168	3.10E−24
COL3A1	Lightyellow	0.481693	0.917229	0.000129	4.76E−24
FAP	Lightyellow	0.522662	0.914703	2.57E−05	1.07E−23
NKX3-2	Lightyellow	0.436338	0.912589	0.000617	2.06E−23
TWIST1	Lightyellow	0.436359	0.906986	0.000617	1.09E−22
WISP1	Lightyellow	0.478241	0.902639	0.000147	3.68E−22
COL1A1	Lightyellow	0.48874	0.896033	9.92E−05	2.11E−21
MATN3	Lightyellow	0.423758	0.893841	0.000917	3.67E−21
COL11A1	Lightyellow	0.506507	0.890533	4.98E−05	8.29E−21
COL10A1	Lightyellow	0.48897	0.889692	9.83E−05	1.02E−20
SRPX2	Lightyellow	0.487384	0.88712	0.000104	1.87E−20
THBS2	Lightyellow	0.500577	0.88698	6.29E−05	1.93E−20
ADAMTS2	Lightyellow	0.40372	0.881852	0.001674	6.23E−20
C3orf80	Lightyellow	0.411638	0.877935	0.001326	1.47E−19
TNFAIP6	Lightyellow	0.480372	0.874937	0.000135	2.79E−19
COL5A2	Lightyellow	0.423757	0.870184	0.000918	7.41E−19
VCAN	Lightyellow	0.457712	0.864524	0.000303	2.26E−18
DRP2	Lightyellow	0.487352	0.85884	0.000105	6.61E−18
MMP13	Lightyellow	0.450368	0.856966	0.000389	9.31E−18
CORIN	Lightyellow	0.506103	0.856206	5.06E-05	1.07E−17
CTHRC1	Lightyellow	0.422339	0.855813	0.000959	1.15E−17
COL1A2	Lightyellow	0.475445	0.851442	0.000162	2.49E−17

*GS, gene significance; MM, module membership.*

### lncRNA/mRNA Network Construction

**FIGURE 7:**
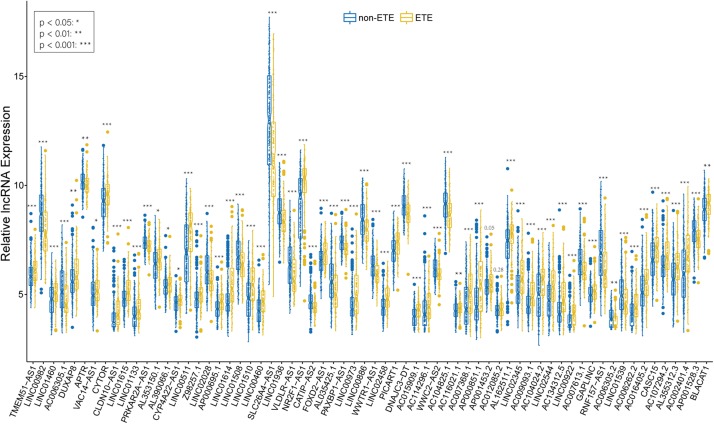
Boxplots of hub mRNA-regulating lncRNAs between ETE and non-ETE samples in the TCGA438 dataset. *p* values were calculated using the Wilcoxon rank sum test. *p < 0.05, **p < 0.01, and ***p < 0.001.

We identified 226 lncRNAs from the salmon and turquoise modules that were significantly relevant to ETE. The TCGA58 dataset was used to correlate these lncRNAs with the hub genes by Pearson correlation analysis, and the results were crossvalidated. Based on the threshold criteria, 66 lncRNAs were significantly coexpressed with 24 hub genes were selected as candidate regulatory lncRNAs (Supplementary Table S5). To validate the associations between these lncRNAs and ETE, the TCGA438 dataset was analyzed for expression differences between ETE and non-ETE samples. The result revealed that 64/66 lncRNAs were significantly differentially expressed (Figure 7) and these were included as regulatory lncRNAs in further analysis.

### TF/lncRNA and TF/mRNA Network Construction

TF prediction analysis resulted in 108 TFs that may regulate 20/33 hub genes, and 153 TFs that may regulate 45/64 regulatory lncRNAs. Correlation analysis revealed that 37/108 TFs showed significant coexpression with 14/20 hub mRNAs, and 110/153 TFs showed significant coexpression with 42/45 regulatory lncRNAs; these were included as regulatory TFs for the mRNAs and lncRNAs, respectively, for a total of 111 unique TFs. The TCGA438 dataset was used to validate the associations between the 111 TFs and ETE, and 64/111 were successfully validated (Supplementary Table S6). After removing the TFs that failed validation, the TF/mRNA network included 30 TFs and 14 hub mRNAs (Supplementary Table S7) and the TF/lncRNA network included 64 TFs and 40 lncRNAs (Supplementary Table S8).

### TF/lncRNA/mRNA Network Construction and Visualization

**FIGURE 8:**
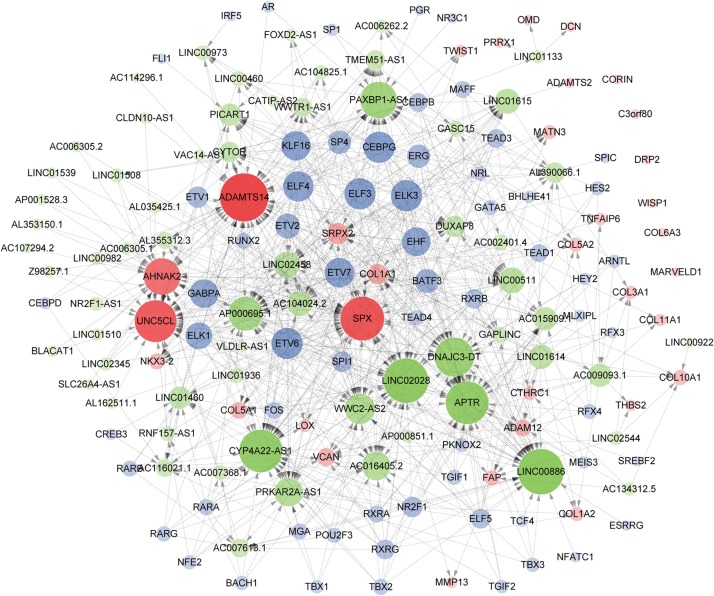
Construction of the TF/lncRNA/mRNA regulatory network. Hub genes, TFs, and lncRNAs are denoted in red, blue, and green, respectively. The point size indicates the node edgecount and the arrow represents regulatory effect.

As a result of the above analyses, a total of 161 genes, including 33 hub mRNAs, 64 lncRNAs, and 64 TFs were used to construct a TF/lncRNA/mRNA regulatory network for ETE. This large network includes three subnetworks: an lncRNA/mRNA regulatory network including 24 mRNAs and 64 lncRNAs, a TF/lncRNA regulatory network including 64 TFs and 40 lncRNAs, and a TF/mRNA regulatory network including 30 TFs and 14 mRNAs (Figure 8).

### Receiver Operating Characteristic and Survival Analyses

**FIGURE 9:**
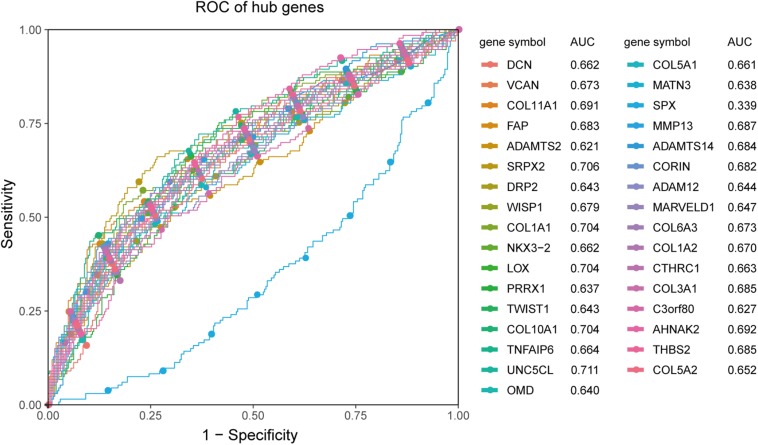
ROC analysis and AUC calculations for hub genes in the TCGA438 dataset. Genes with AUC values <0.5 were considered downregulated. The greater the value of |AUC-0.5|, the more meaningful the hub gene is for ETE diagnosis.

**FIGURE 10:**
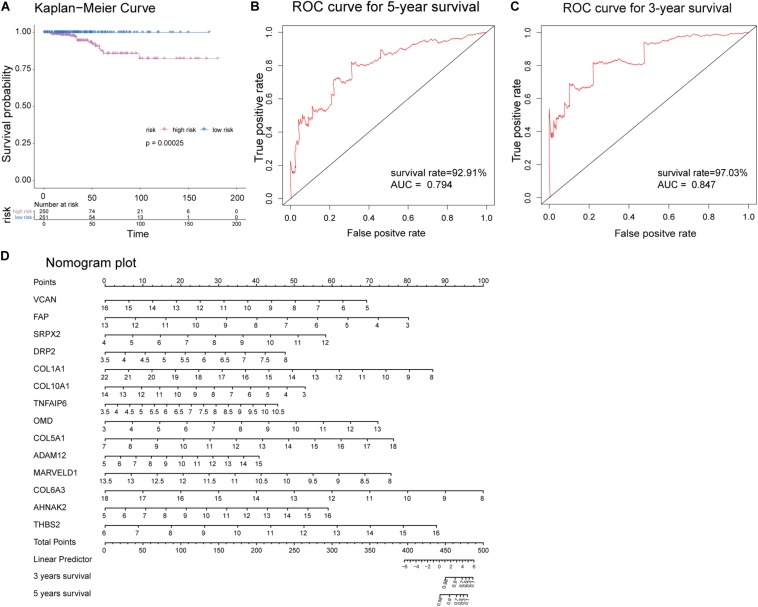
Evaluation of the optimized Cox proportional hazards model effectiveness. **(A)** Kaplan-Meier survival curves of high- and low-risk groups based on the median riskscores. **(B,C)** Time-dependent receiver operating characteristic (ROC) was used to evaluate the performance of Cox regression prognostic models for 3- and 5-year overall survival. **(D)** Nomogram plot was constructed with the 14 hub genes for overall survival. The effects of each hub genes can be converted into the point’s axis at the top of the nomogram. After adding the points of each hub genes and corresponding to the total point’s axis at the bottom of the nomogram, a patient’s probability of survival (3- and 5-year) can be predicted at the bottom of the nomogram.

Receiver operating characteristic analysis and AUC calculations for the 33 hub genes are shown in Figure 9. The average AUC of the hub genes was 0.67, and unc-5 family C-terminal like (AUC: 0.711), sushi repeat containing protein X-linked 2 (AUC: 0.706), lysyl oxidase (AUC: 0.704), collagen (COL) type I alpha 1 chain (AUC: 0.704), and COL type X alpha 1 chain (AUC: 0.704) were the most highly significant genes predicting ETE diagnosis in PTC. So the results indicated that these genes may be used as candidates for the clinical diagnosis of ETE. Survival analysis demonstrated that most of the hub genes could influence the survival prognosis of patients with PTC to some extent, but only one gene, the serine protease corin, was statistically significant (*p* = 0.018; Supplementary Figure S4). Considering these hub genes may affect the prognosis through a cooperative mechanism, and the role of an individual hub gene was not so significant. Therefore, we try to further clarify the effect of hub genes on the survival prognosis of PTC. One-way ANOVA analysis demonstrated age (*p* = 5.9E−9), tumor_size (*p* = 0.02), extrathyroid_extension (*p* = 0.045), pathologic_M (*p* = 0.032), and pathologic_stage (*p* = 0.0001) were significant influential factors for the survival prognosis of PTC patients (Supplementary Table S9). Then, we constructed a multivariable Cox proportional hazards model, and the optimized Cox regression model using the AIC algorithm contained 14 hub genes at last (Supplementary Table S10) and the total 501 PTC patients were classified into high- and low-risk groups based on the median of the risk scores 0.923 (Supplementary Table S11). The result of the Kaplan-Meier curves and log-rank test showed a significant difference between the high- and low-risk groups (*p* = 0.00025) (Figure 10A). This suggested that these hub genes are statistically significant in predicting patient prognosis. Besides, the time-dependent ROC showed that AUC for 3- and 5-year OS was calculated separately as 0.847 (95% CI: 0.701–0.964) and 0.794 (95% CI: 0.714–0.896), and C-index was calculated as 0.895 (95% CI: 0.809–0.952), both indicating the Cox regression model performed good prediction for PTC survival prognosis (Figures 10B,C, Supplementary Figure S5). The nomogram was developed based on the results of the multivariable Cox proportional hazards model. A weighted score calculated using the 14 hub genes was used to estimate 3- and 5-year OS (Figure 10D).

## Discussion

In this study, we identified three gene modules (lightyellow, greenyellow, and brown) and two lncRNA modules (salmon and turquoise) that were significantly related to ETE in PTC. Among the three gene modules, 33 genes were screened and validated as hub genes that may play important roles in ETE development and progression. Through correlation analysis and TF prediction, we have constructed a transcriptional regulatory network for ETE, which provides a myriad of clues toward the elucidation of the molecular mechanisms of ETE in PTC.

It is widely recognized that ETE is a major factor affecting PTC prognosis, and the presence of ETE is an important staging consideration for PTC, making understanding its molecular mechanisms and identifying biomarkers important. In previous studies, Chakraborty et al. (2012) and Lee et al. (2017) found that B-Raf proto-oncogene mutation status was significantly related to extrathyroidal invasion and lymph node metastasis. However, no studies identifying biomarkers of ETE in PTC have been reported.

The tumor microenvironment encompasses tumor cells and the tumor stroma, such as cancer-associated fibroblasts, endothelial cells, immune cells, cancer-associated adipocytes, and the ECM (Hanahan and Coussens, 2012; Bussard et al., 2016; Brioschi and Colonna, 2019), and plays an important role in tumor progression through the interaction and co-evolution of tumor cells and the stroma (Garcia et al., 2011; Fang et al., 2014; Wu et al., 2019). Interestingly, functional annotation revealed that all three ETE-related gene modules were enriched in genes involved in the regulation of ECM. The ECM, which is mainly composed of collagen, fibronectin, immune cells, proteoglycans, and glycosaminoglycans, is the main component of tumor stroma and a key regulator of cell and tissue functions. The results suggest that dynamic changes in the tumor microenvironment, and particularly in the ECM, play important roles in ETE.

The lightyellow module was the module most significantly associated with ETE. GO analysis revealed enrichment in biological processes involved in ECM organization, which can involve the assembly, rearrangement, or disassembly of the ECM. It is worth noting that ECM remodeling affects cell adhesion, tumor migration, and angiogenesis by triggering biochemical and biophysical signals (Paszek et al., 2005; Butcher et al., 2009; Wolf and Friedl, 2009). ECM remodeling is tightly controlled in normal tissues but is usually dysregulated in cancer (Page-McCaw et al., 2007). In addition, KEGG analysis indicated enrichment in many cancer-associated pathways, including those involved in aspects of ECM remodeling, and interestingly, the phosphoinositide 3-kinase/Akt pathway, which plays important roles in transformation and in cancer cell cycle progression and apoptosis (Chang et al., 2003). These results suggest that ECM remodeling is closely related to the development of ETE.

In the brown module, GO analysis suggested functions related to the immune response. Immune cells are an important component of the tumor stroma and immune-mediated dysregulation has been associated with many cancers (Balkwill et al., 2005; de Visser et al., 2006; Wyckoff et al., 2007; Bergdorf et al., 2019). Consistently, KEGG pathway analysis also suggested immunoregulatory functions, as well as enrichment in cancer-associated pathways. Therefore, genes in the brown module may participate in ETE development through immune-mediated dysregulation.

For the greenyellow module, GO analysis identified enrichment in genes related to the ECM, as in the lightyellow module. However, KEGG pathway analysis identified enrichment in multiple signal transduction pathways. Signal transduction between the ECM and cells plays an important role in the dynamic changes of ECM biomechanics associated with tumor progression (Essex et al., 2001; Hoffman et al., 2011). Hence, genes in the greenyellow module may promote ETE development by transducing signals from the tumor microenvironment.

In these ETE-related gene modules, we identified 33 hub genes that may serve as important biomarkers for ETE and have important clinical implications for improving risk stratification, treatment decision-making, and prognosis prediction in patients with PTC. Interestingly, most of the products of these genes are ECM components, including eight hub genes (*COL3A1*, *COL6A3*, *COL1A2*, *COL1A1*, *COL11A1*, *COL5A2*, *COL5A1*, and *COL10A1*) that encode collagen. As the most abundant component of the ECM, collagen is crucial for ECM function, and both increased and decreased collagen deposition are involved in biomechanical force changes associated with tumor invasion and migration (Butcher et al., 2009; Levental et al., 2009). The hub gene lysyl oxidase encodes an ECM protein that increases insoluble matrix deposition and tissue stiffness by crosslinking collagens with elastin, and is essential to enable tumor cells to escape from primary sites and grow at secondary sites during metastasis (Wang et al., 2016; Johnston and Lopez, 2018). Collagen degradation is necessary for tumor invasion, and matrix metalloproteinases, which play important roles in this process, have direct causative effects on tumor progression (Page-McCaw et al., 2007; Hoffman et al., 2011; Torzilli et al., 2012). The hub gene matrix metalloproteinase 13 promotes the progression of different kinds of cancers (Dumortier et al., 2018; Liu et al., 2018). Therefore, these hub genes may promote the progression of ETE in PTC through direct or indirect interactions.

As known, ETE was one of the most important factors affecting the prognosis of PTC patients and whether there is ETE will determine different treatment strategies such as different surgery methods and whether combining with postoperative radiotherapy and so on. Therefore, we speculate that these hub genes closely related to ETE could serve as biomarkers for ETE diagnosis and affect survival prognosis to an extent. To verify this idea, we first performed Wilcoxon rank sum test and ROC analysis, the results showed each hub gene was able to distinguish ETE and non-ETE samples and performed a relative good prediction in ETE diagnosis. Then KM curves and log-rank test showed most hub genes could affect PTC survival prognosis to an extent, but only one hub gene was statistically significant. Possible reasons leading to this result include insufficient sample size, especially for the sample numbers of dead patients, and the interference of other factors affecting survival prognosis, such as age and metastasis. Considering that these hub genes may influence the prognosis through a mechanism that works together, and the role of individual genes was not so significant. Therefore, we constructed a multivariable Cox proportional hazards model using hub genes and survival information to further clarify the effect of hub genes on the survival prognosis of PTC. Survival analysis for the high- and low-risk groups showed hub genes are statistically significant in predicting patient prognosis. Besides, the time-dependent ROC and C-index for checking the accuracy of the Cox regression model both suggested good prediction ability. The time-dependent ROC showed the predicted 3- and 5-year survival rate was 97.03 and 92.91% separately, which were consistent with previous studies (Kitahara et al., 2017; Liu et al., 2017). In the nomogram plot, weighted scores calculated using the 14 hub genes were used to estimate 3- and 5-year OS and that means we could use these hub genes and weighted scores to predict the OS of PTC patients, but still need large amounts of clinical data to validate. In summary, as we explored the relationship of 33 hub genes to ETE diagnosis and PTC survival prognosis, we found the 33 hub genes closely associated with ETE demonstrated relative good prediction in ETE diagnosis. In addition, selected 14 hub genes constructing the Cox regression model could perform a good prediction for OS of PTC patients.

Understanding the regulation of gene expression is of fundamental importance, as the vast majority of biological processes are regulated by differential gene expression. It has been demonstrated that lncRNAs, non-coding RNA >200 nucleotides in length, can regulate gene expression through epigenetic, transcriptional, posttranscriptional, and translational mechanisms, and play important roles in cancer development (Schmitz et al., 2016; Sun et al., 2016; Li et al., 2019; Zhang et al., 2019). Since the three mRNA modules and two lncRNA modules are all closely related to ETE, we speculated that genes in these modules have regulatory relationships. Therefore, we constructed a lncRNA/mRNA regulatory network including 24 hub mRNAs and 64 lncRNAs. TFs are DNA-binding proteins whose gene regulation ability is crucial to define the molecular state of cells (Simicevic and Deplancke, 2017). Therefore, predicting TFs for these genes was crucial to construct a complete regulatory network. TF binding motifs dictate the binding specificity of gene promoters or cis-regulatory modules, usually by pooling a series of conserved and variable binding sites (Harbison et al., 2004; Maston et al., 2006). Our regulatory network includes 64 TFs and provides new insights into the molecular mechanisms of ETE.

In conclusion, through comprehensive bioinformatics analysis, this study identified 33 biomarkers and multiple signaling pathways related to ETE in PTC, resulting in a transcriptional regulatory network for ETE including TFs, lncRNAs, and mRNAs. Moreover, ROC and survival analysis showed these hub genes could serve as candidates for ETE diagnosis and prognosis of PTC patients. Verification with a larger number of samples will be required; however, currently few datasets with complete ETE information exist. Also, our results will require lots of experimental verification. Taken together, our findings provide novel insights into the molecular mechanisms of ETE in PTC, and may have important clinical implications in the improvement of PTC risk stratification, therapeutic decision-making, and prognosis prediction.

## Data Availability Statement

The RNA-seq dataset containing 568 samples from patients with TC can be found in the TCGA database (https://portal.gdc.cancer.gov). The matched clinical data can be obtained from UCSC Xena (http://xena.ucsc.edu/). The RNA-seq datasets (GSE83520 and GSE64912) can be found in the GEO database (https://www.ncbi.nlm.nih.gov/geo/query/acc.cgi?acc=GSE83520 and https://www.ncbi.nlm.nih.gov/geo/query/acc.cgi?acc=GSE64912). The microassay datasets (GSE33630 and GSE60542) can be found in the GEO database (https://www.ncbi.nlm.nih.gov/geo/query/acc.cgi?acc=GSE33630 and https://www.ncbi.nlm.nih.gov/geo/query/acc.cgi?acc=GSE60542). The gene expression matrix (log2 fragments per kilobase of transcript per million mapped reads) of 568 TC samples can be obtained from UCSC Xena (http://xena.ucsc.edu/). The human reference genome data (version: GRCh38.p12) and related human binding motif data can be downloaded from the Ensemble BioMart database (http://asia.ensembl.org/index.html).

## Author Contributions

YC and XL conceived and designed the study. YC, BJ, and WW downloaded the datasets and performed the analysis procedures. YC, DS, and FX analyzed the results. YC and XL were involved in writing and editing the manuscript.

## Conflict of Interest

The authors declare that the research was conducted in the absence of any commercial or financial relationships that could be construed as a potential conflict of interest.

## Footnotes

1 https://portal.gdc.cancer.gov

2 http://xena.ucsc.edu/

3 http://www.ncbi.nlm.nih.gov/geo/

4 http://asia.ensembl.org/index.html

5 http://gepia.cancer-pku.cn/detail.php
