# Supplementary material for: Identifying the Transcriptional Regulatory Network Associated With Extrathyroidal Extension in Papillary Thyroid Carcinoma by Comprehensive Bioinformatics Analysis
Source: Front Genet. 2020 May 11;11:453. doi: 10.3389/fgene.2020.00453 (PMC7232969; doi:10.3389/fgene.2020.00453)
Supplement: Supplementary file 1 [file Table_1.DOCX]

**The specific process for our team to predict the transcription factor of the target genes is as follows：**

(1) Document preparation：First download the Human genes (GRCh38.p12) and Human Binding Motifs (GRCh38.p12) through the ENSEMBLE BioMart database (<http://useast.ensembl.org/biomart/martview/e3cabe89d6d5ff5876c4a2ca242fd7a7>)，and then rename them GRCh38.gene.bed and GRCh38.TFmotif_binding.bed respectively. Then prepare the target gene list, named mRNA.list and lncRNA.list respectively.

(2) File format processing:

sed -i '1d' GRCh38.gene.bed

sed -i 's/-1$/-/' GRCh38.gene.bed

sed -i 's/1$/+/' GRCh38.gene.bed

sed -i 's/^/chr/' GRCh38.gene.bed

sed -i ‘/::/d’ GRCh38.TFmotif_binding.bed

(3) Calculating the promoter regions of genes（The promoter region of a gene was defined as the 1,000 bp upstream and 200 bp downstream of the transcriptional start site）:

awk 'BEGIN{OFS=FS="\t"}{if($6=="+") {tss=$2; tss_up=tss-1000; tss_dw=tss+200;} else {tss=$3; tss_up=tss-200; tss_dw=tss+1000;} if(tss_up<0) tss_up=0;print $1, tss_up, tss_dw,$4,$5,$6;}' GRCh38.gene.bed > GRCh38.gene.promoter.U1000D200.bed

(4) Predict the target genes by known TF binding motifs:

bedtools intersect -a GRCh38.gene.promoter.U1000D200.bed -b GRCh38.TFmotif_binding.bed -wa -wb | cut -f 5,11 | sort -u | tr 'a-z' 'A-Z' > GRCh38.gene.promoter.U1000D200.TF_binding.txt

(5) Extract the list of genes we care about:

awk 'BEGIN{OFS=FS="\t"}ARGIND==1{save[$1]=1}ARGIND>1{if(save[$1]==1) {print $0}}' mRNA.list GRCh38.gene.promoter.U1000D200.TF_binding.txt > mRNA.TF_bing.txt

awk 'BEGIN{OFS=FS="\t"}ARGIND==1{save[$1]=1}ARGIND>1{if(save[$1]==1) {print $0}}' lncRNA.list GRCh38.gene.promoter.U1000D200.TF_binding.txt > lncRNA.TF_bing.txt
