# Supplementary material for: Identifying the Transcriptional Regulatory Network Associated With Extrathyroidal Extension in Papillary Thyroid Carcinoma by Comprehensive Bioinformatics Analysis
Source: Front Genet. 2020 May 11;11:453. doi: 10.3389/fgene.2020.00453 (PMC7232969; doi:10.3389/fgene.2020.00453)
Supplement: Supplementary file 2 [file Table_2.DOCX]

**Supplementary Table S2. Detailed information of datasets used in this study.**

| Dataset | TCGA58 | TCGA438 | GSE33630 | GSE60542 | GSE64912 | GSE83520 | Expression matrix(FPKM) |
| --- | --- | --- | --- | --- | --- | --- | --- |
| Platform | IlluminaHiseq | IlluminaHiseq | Affymetrix HG  U133 Plus 2.0 | Affymetrix HG  U133 Plus 2.0 | IlluminaHiseq | IlluminaHiseq | IlluminaHiseq |
| Total samples | 116 | 438 | 94 | 63 | 22 | 24 | 509 |
| Tumor | 58 | 438 | 49 | 33 | 18 | 12 | 501 |
| ETE | 23 | 133 | - | - | - | - | - |
| non-ETE | 35 | 305 | - | - | - | - | - |
| Normal | 58 | 0 | 45 | 30 | 4 | 12 | 58 |

TCGA58: contains tumor samples and matched healthy samples from 58 patients with PTC.

TCGA438: contains 438 PTC tumor samples.

GSE33630: contains 49 PTC tumor samples and 45 normal samples.

GSE60542: contains 33 PTC tumor samples and 30 normal samples.

GSE64912: contains 18 PTC tumor samples and 4 normal samples.

GSE83520: contains 49 PTC tumor samples and 45 normal samples.

Expression matrix (FPKM): contains 501 PTC tumor samples and 58 normal samples.
