# Supplementary material for: Identifying the Transcriptional Regulatory Network Associated With Extrathyroidal Extension in Papillary Thyroid Carcinoma by Comprehensive Bioinformatics Analysis
Source: Front Genet. 2020 May 11;11:453. doi: 10.3389/fgene.2020.00453 (PMC7232969; doi:10.3389/fgene.2020.00453)
Supplement: Supplementary file 6 [file Table_6.DOCX]

**Supplementary Table S6. Wilcoxon rank sum test for TFs**

| Gene symbol | wilcox.test.p |  |  | Gene symbol | wilcox.test.p |
| --- | --- | --- | --- | --- | --- |
| EHF | 2.69E-09 |  |  | CEBPD | 0.017912 |
| ELF3 | 3.62E-09 |  |  | MLXIPL | 0.020664 |
| RFX4 | 6.44E-08 |  |  | NR3C1 | 0.023625 |
| BACH1 | 7.34E-08 |  |  | SP4 | 0.024084 |
| NRL | 1.35E-07 |  |  | NFE2 | 0.02567 |
| RARA | 2.32E-07 |  |  | TBX1 | 0.025724 |
| NR2F1 | 3.47E-07 |  |  | TCF4 | 0.026497 |
| PKNOX2 | 4.94E-07 |  |  | RXRB | 0.027404 |
| ELK3 | 7.12E-07 |  |  | SPIC | 0.03407 |
| ARNTL | 9.55E-07 |  |  | RFX5 | 0.055896 |
| KLF16 | 1.84E-06 |  |  | USF1 | 0.056319 |
| POU2F3 | 2.94E-06 |  |  | ETV5 | 0.068524 |
| HES2 | 3.22E-06 |  |  | TBX4 | 0.082947 |
| RARG | 4.24E-06 |  |  | TCF3 | 0.084437 |
| PGR | 4.32E-06 |  |  | RUNX3 | 0.097279 |
| RUNX2 | 6.90E-06 |  |  | NR3C2 | 0.100128 |
| MAFF | 1.27E-05 |  |  | ELK4 | 0.11331 |
| CEBPG | 3.25E-05 |  |  | ESRRA | 0.115003 |
| TEAD1 | 3.97E-05 |  |  | ETV4 | 0.154616 |
| ELK1 | 4.29E-05 |  |  | MEIS2 | 0.159428 |
| RARB | 4.69E-05 |  |  | MEF2D | 0.159915 |
| ESRRG | 4.99E-05 |  |  | SP3 | 0.167868 |
| BHLHE41 | 7.05E-05 |  |  | ATF7 | 0.177165 |
| ETV2 | 8.63E-05 |  |  | NR2F6 | 0.18684 |
| ELF4 | 0.000108 |  |  | JUN | 0.18684 |
| TBX2 | 0.000112 |  |  | ELF1 | 0.187115 |
| TGIF1 | 0.000112 |  |  | ETS1 | 0.190151 |
| GABPA | 0.000127 |  |  | E2F1 | 0.235876 |
| BATF3 | 0.000221 |  |  | MAFG | 0.294892 |
| TGIF2 | 0.000249 |  |  | TFEB | 0.302526 |
| ETV7 | 0.000257 |  |  | MEF2A | 0.334364 |
| AR | 0.000375 |  |  | CEBPE | 0.352126 |
| RFX3 | 0.000715 |  |  | JDP2 | 0.354894 |
| TEAD3 | 0.000752 |  |  | CLOCK | 0.383328 |
| GATA5 | 0.000846 |  |  | IRF8 | 0.43259 |
| IRF5 | 0.001306 |  |  | ELF2 | 0.450613 |
| RXRA | 0.001954 |  |  | TFEC | 0.452092 |
| CEBPB | 0.002711 |  |  | TBX15 | 0.467039 |
| ERG | 0.00352 |  |  | MLX | 0.470061 |
| FLI1 | 0.003981 |  |  | POU2F1 | 0.478172 |
| TBX3 | 0.006266 |  |  | IRF2 | 0.497219 |
| ELF5 | 0.006281 |  |  | ETV3 | 0.501912 |
| SP1 | 0.006851 |  |  | TBX5 | 0.515846 |
| SREBF2 | 0.007005 |  |  | PKNOX1 | 0.532711 |
| CREB3 | 0.007746 |  |  | E2F3 | 0.570557 |
| SPI1 | 0.008577 |  |  | E2F4 | 0.582887 |
| NFATC1 | 0.008681 |  |  | CTCF | 0.652822 |
| ETV1 | 0.009855 |  |  | ATF4 | 0.757586 |
| FOS | 0.010408 |  |  | GATA3 | 0.769479 |
| MEIS3 | 0.011488 |  |  | EBF1 | 0.79406 |
| TEAD4 | 0.012433 |  |  | E2F2 | 0.822039 |
| RXRG | 0.014534 |  |  | HES7 | 0.916643 |
| HEY2 | 0.015627 |  |  | SPIB | 0.91762 |
| ETV6 | 0.016605 |  |  | FOXC1 | 0.9398 |
| MGA | 0.017363 |  |  | MYBL2 | 0.991486 |
