# Supplementary material for: Identifying the Transcriptional Regulatory Network Associated With Extrathyroidal Extension in Papillary Thyroid Carcinoma by Comprehensive Bioinformatics Analysis
Source: Front Genet. 2020 May 11;11:453. doi: 10.3389/fgene.2020.00453 (PMC7232969; doi:10.3389/fgene.2020.00453)
Supplement: Supplementary file 9 [file Table_9.DOCX]

TaBle 1. Clinical Characteristics of PTC patients from TCGA (n = 501)

|  |  |  | Beta | HR | 95% CI | wald.test | *p.value |
| --- | --- | --- | --- | --- | --- | --- | --- |
| gender | female | 366 | 0.67 | 2 | (0.71-5.4) | 1.7 | 0.19 |
|  | male | 135 |  |  |  |  |  |
| age | Mean(years) | 47.33 | 0.11 | 1.1 | (1.1-1.2) | 34 | 5.90E-09 |
| tumor_size | Mean(mm) | 2.5 | 0.3 | 1.3 | (1-1.7) | 5.3 | 0.022 |
|  | NA | 14 |  |  |  |  |  |
| tumor_focus_type | Unifocal | 265 | -1.4 | 0.25 | (0.057-1.1) | 3.2 | 0.072 |
|  | Multifocal | 226 |  |  |  |  |  |
|  | NA | 10 |  |  |  |  |  |
| extrathyroid_extension | No | 340 | 1 | 2.8 | (1-7.8) | 4 | 0.045 |
|  | Yes | 156 |  |  |  |  |  |
|  | NA | 5 |  |  |  |  |  |
| pathologic_N | N0 | 278 | 0.14 | 1.1 | (0.43-3.1) | 0.08 | 0.78 |
|  | N1 | 223 |  |  |  |  |  |
| pathologic_M | M0 | 491 | 1.6 | 5.1 | (1.1-23) | 4.6 | 0.032 |
|  | M1 | 9 |  |  |  |  |  |
|  | NA | 1 |  |  |  |  |  |
| pathologic_stage | stage i | 281 | 0.93 | 2.5 | (1.6-4) | 15 | 0.0001 |
|  | stage ii | 51 |  |  |  |  |  |
|  | stage iii | 112 |  |  |  |  |  |
|  | stage iv | 55 |  |  |  |  |  |
|  | NA | 2 |  |  |  |  |  |

* by one way ANOVA analysis and p<0.05 was considered to be significant

Abbreviations: NA, not available; PTC, papillary thyroid cancer; HR, hazard ratios; 95% CI, 95% confidence intervals.
