# Supplementary material for: Identifying the Transcriptional Regulatory Network Associated With Extrathyroidal Extension in Papillary Thyroid Carcinoma by Comprehensive Bioinformatics Analysis
Source: Front Genet. 2020 May 11;11:453. doi: 10.3389/fgene.2020.00453 (PMC7232969; doi:10.3389/fgene.2020.00453)
Supplement: Supplementary file 10 [file Table_10.DOCX]

Supplementary Table S10:

The optimized Cox proportional hazards model using AIC with 14 hub genes

| Gene symbol | coef | exp(coef) | se(coef) | z | Pr(>\|z\|) |
| --- | --- | --- | --- | --- | --- |
| VCAN | -0.8205 | 0.4402 | 0.5044 | -1.627 | 0.10382 |
| FAP | -1.0475 | 0.3508 | 0.5714 | -1.833 | 0.06676 |
| SRPX2 | 0.9516 | 2.5898 | 0.5196 | 1.831 | 0.06705 |
| DRP2 | 1.3807 | 3.9777 | 0.5098 | 2.708 | 0.00676 |
| COL1A1 | -0.8077 | 0.4459 | 0.4359 | -1.853 | 0.06387 |
| COL10A1 | -0.6277 | 0.5338 | 0.2983 | -2.104 | 0.03537 |
| TNFAIP6 | 0.8507 | 2.3412 | 0.4848 | 1.755 | 0.07933 |
| OMD | 0.9416 | 2.564 | 0.2931 | 3.212 | 0.00132 |
| COL5A1 | 0.9049 | 2.4716 | 0.394 | 2.297 | 0.02163 |
| ADAM12 | 0.5314 | 1.7012 | 0.3452 | 1.539 | 0.12374 |
| MARVELD1 | -1.7937 | 0.1663 | 0.65 | -2.759 | 0.00579 |
| COL6A3 | -1.3051 | 0.2712 | 0.5281 | -2.471 | 0.01347 |
| AHNAK2 | 0.6997 | 2.0132 | 0.2634 | 2.656 | 0.00791 |
| THBS2 | 1.1436 | 3.1379 | 0.6424 | 1.78 | 0.07504 |
