# Supplementary material for: Identifying the Transcriptional Regulatory Network Associated With Extrathyroidal Extension in Papillary Thyroid Carcinoma by Comprehensive Bioinformatics Analysis
Source: Front Genet. 2020 May 11;11:453. doi: 10.3389/fgene.2020.00453 (PMC7232969; doi:10.3389/fgene.2020.00453)
Supplement: Supplementary file 12 [file Data_Sheet_1.PDF]

Supplementary Figure S1

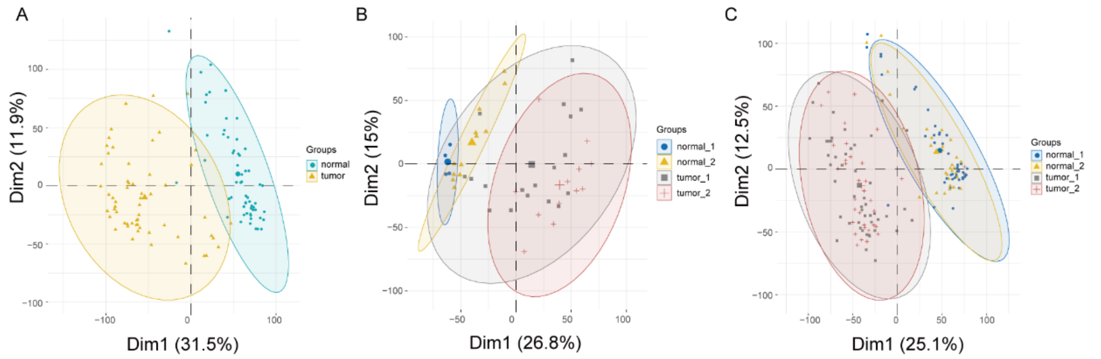

Supplementary Figure S1：Principal component analysis was used to verify dataset quality after data preprocessing. (A) Principal component plot of the dataset TCGA58 showed separation between tumor and normal samples. (B) Principal component plot of the combined dataset GSE64912 and GSE83520 showed overlap between samples in each of the tumor or normal groups and separation between tumor and normal samples. (C) Principal component plot of the combined dataset GSE33630 and GSE60542 showed overlap between samples in each of tumor or normal groups and separation between tumor and normal samples.
