# Supplementary material for: Identifying the Transcriptional Regulatory Network Associated With Extrathyroidal Extension in Papillary Thyroid Carcinoma by Comprehensive Bioinformatics Analysis
Source: Front Genet. 2020 May 11;11:453. doi: 10.3389/fgene.2020.00453 (PMC7232969; doi:10.3389/fgene.2020.00453)
Supplement: Supplementary file 13 [file Data_Sheet_2.PDF]

Supplementary Figure S2

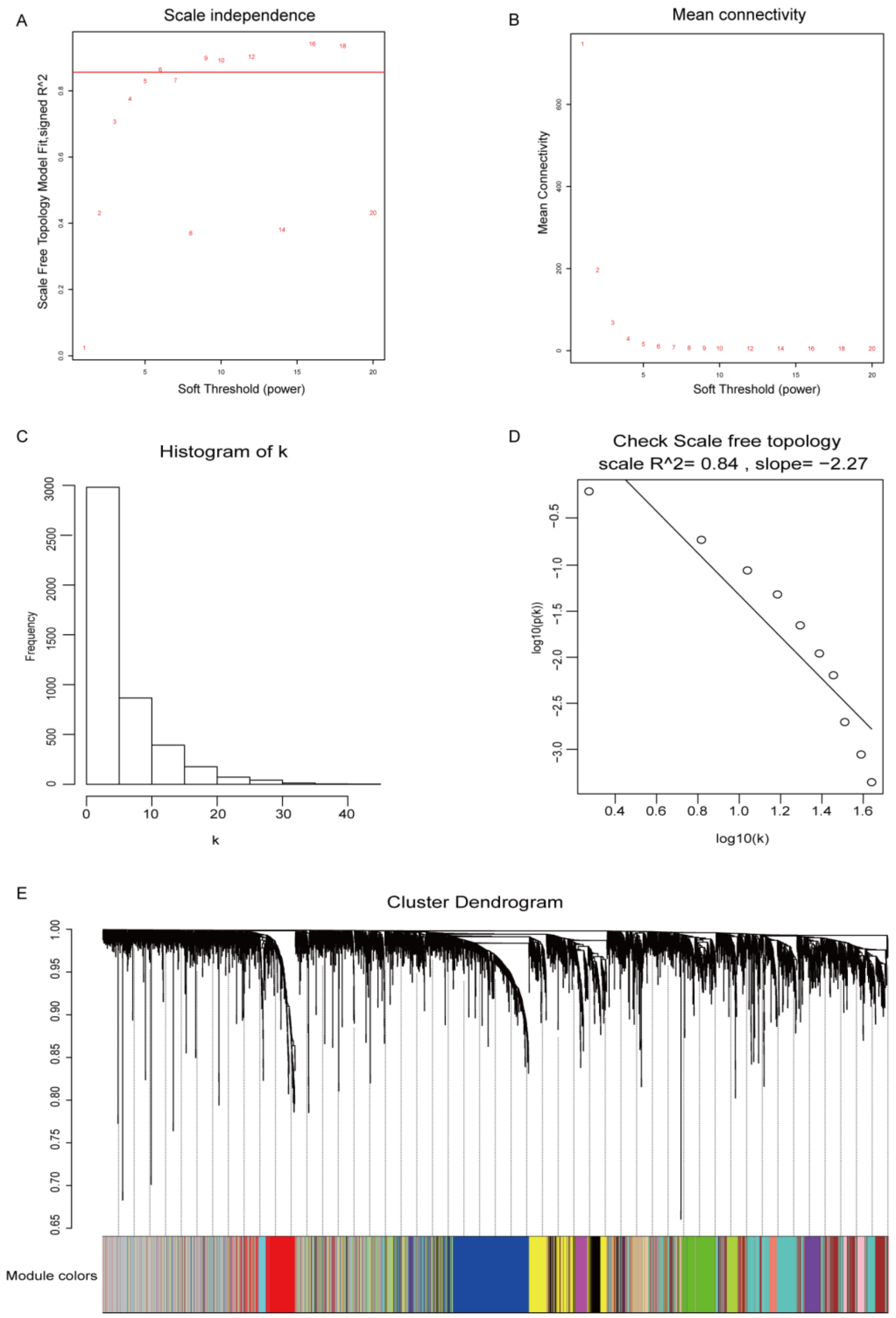

Supplementary Figure S2: DEL weighted gene co-expression network construction. (A-B) The scale-free fit index and the mean connectivity with various soft-thresholding powers. (C-D) Histogram of connectivity distributions and check scale-free topology when $\beta = 6$. (E) DEL clustering dendrograms.
