# Supplementary material for: Identifying the Transcriptional Regulatory Network Associated With Extrathyroidal Extension in Papillary Thyroid Carcinoma by Comprehensive Bioinformatics Analysis
Source: Front Genet. 2020 May 11;11:453. doi: 10.3389/fgene.2020.00453 (PMC7232969; doi:10.3389/fgene.2020.00453)
Supplement: Supplementary file 14 [file Data_Sheet_3.PDF]

**Supplementary Figure S3**

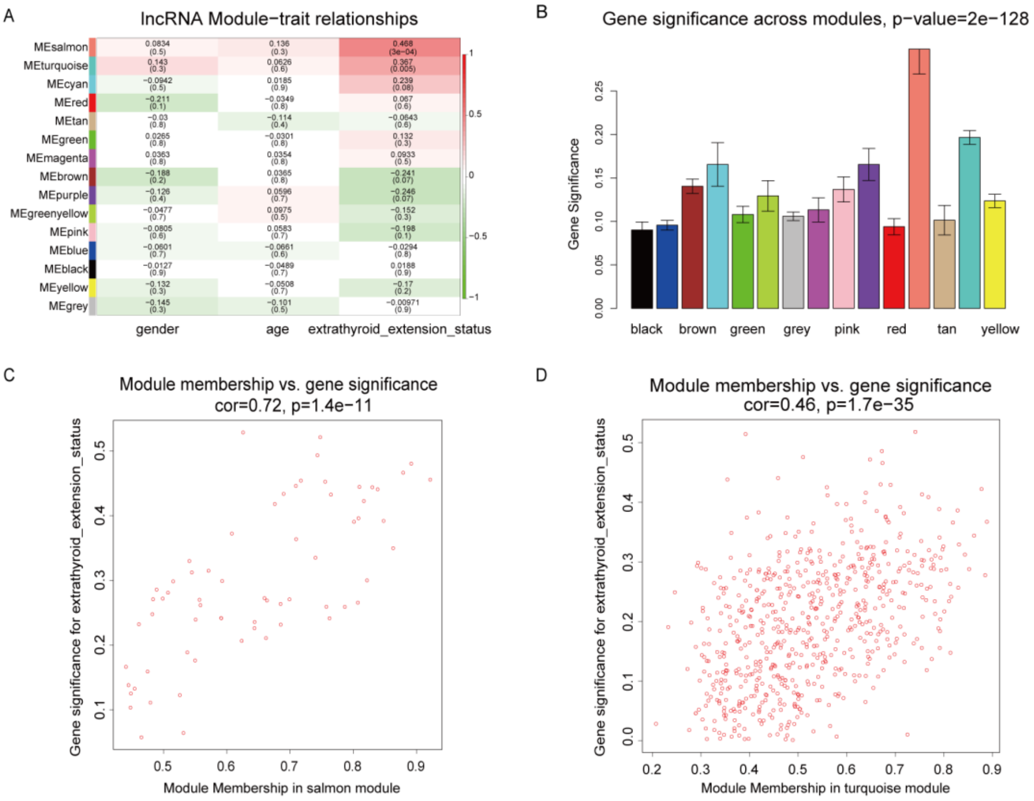


Supplementary Figure S3: Identification of lncRNA modules associated with clinical traits of ETE. (A) Heatmap of module-trait relationships. (B) Distribution of average gene significance in modules related to ETE. (C-D) Scatter plots of correlations between gene module membership and gene significance in the two ETE-related lncRNA modules.
