# Supplementary material for: Identifying the Transcriptional Regulatory Network Associated With Extrathyroidal Extension in Papillary Thyroid Carcinoma by Comprehensive Bioinformatics Analysis
Source: Front Genet. 2020 May 11;11:453. doi: 10.3389/fgene.2020.00453 (PMC7232969; doi:10.3389/fgene.2020.00453)
Supplement: Supplementary file 15 [file Data_Sheet_4.PDF]

Supplementary Figure S4

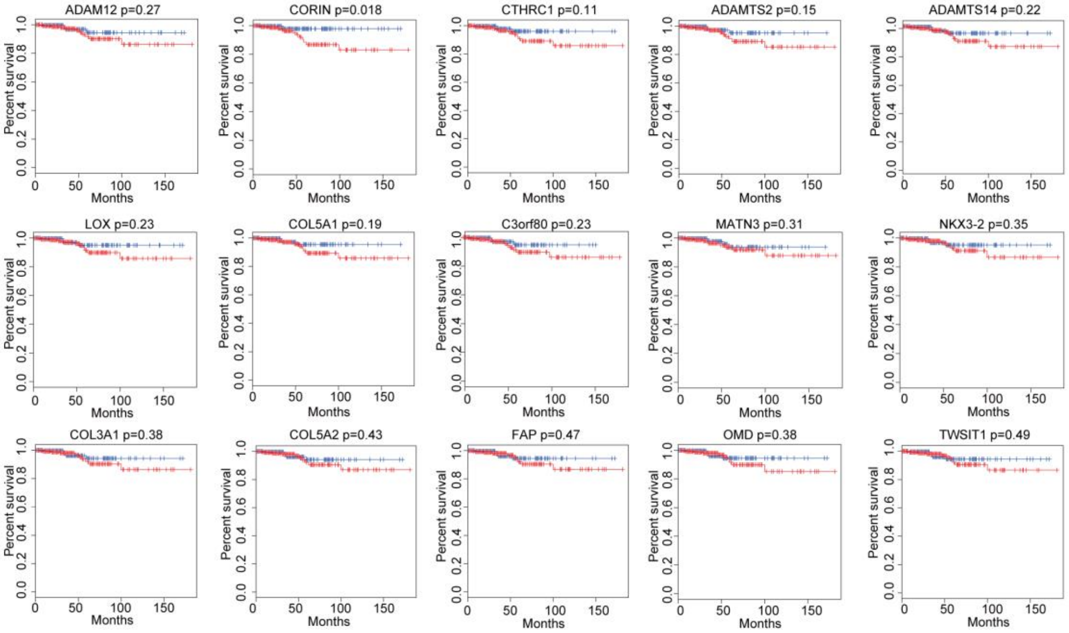

Supplementary Figure S4: Survival analysis for hub genes. Survival curves of the top 15 hub genes are displayed. Red and blue lines represent high and low hub gene expression, respectively.
