# Supplementary material for: Identifying the Transcriptional Regulatory Network Associated With Extrathyroidal Extension in Papillary Thyroid Carcinoma by Comprehensive Bioinformatics Analysis
Source: Front Genet. 2020 May 11;11:453. doi: 10.3389/fgene.2020.00453 (PMC7232969; doi:10.3389/fgene.2020.00453)
Supplement: Supplementary file 16 [file Data_Sheet_5.PDF]

Supplementary Figure S5

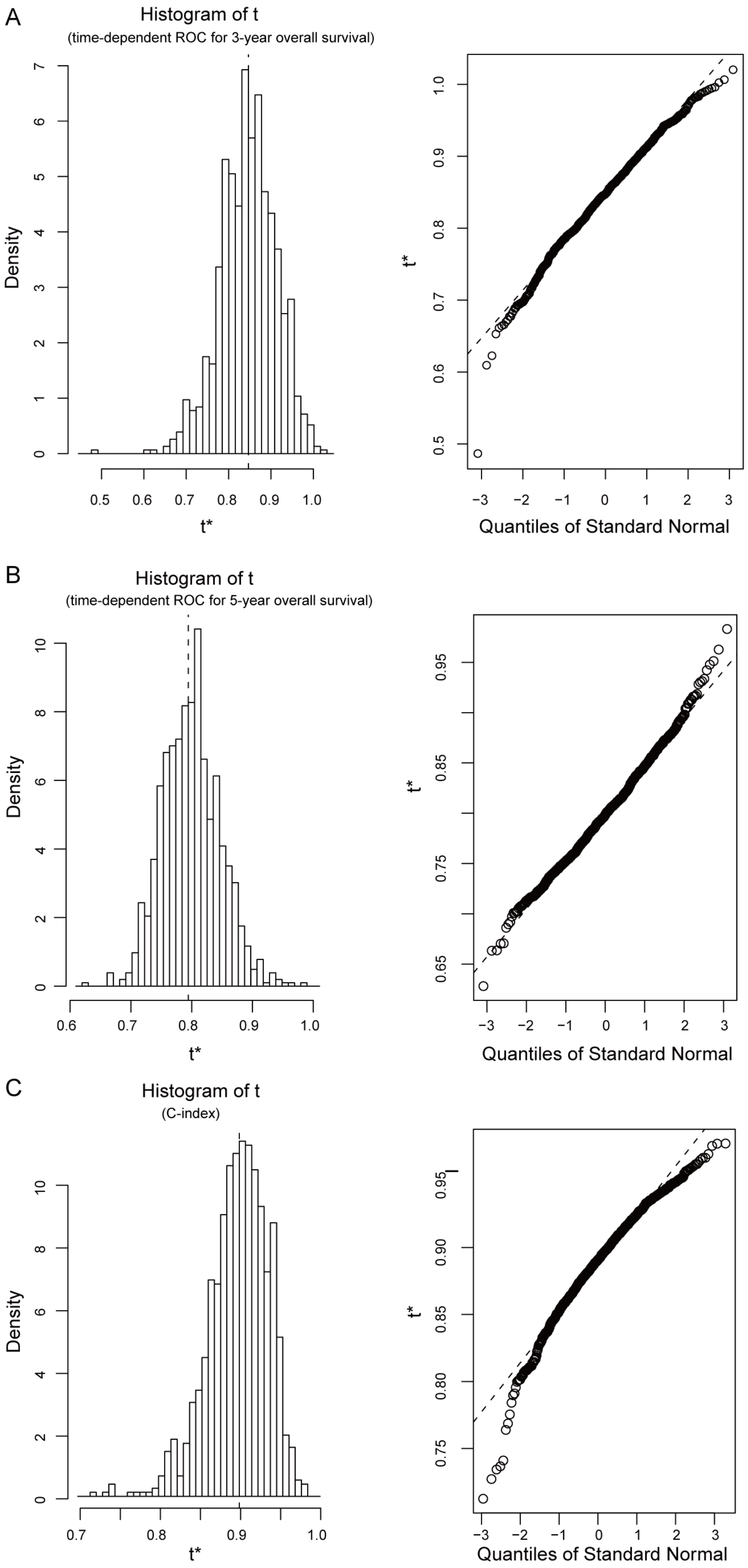

Supplementary Figure S5：Time-dependent ROC (AUC) and C-index of the Cox regression prediction model with 14 hub genes were compared using bootstrap with 1000 times re-sampling separately. (A) Histogram and fitted curve with AUC values repeated 1000 times for 3-year overall survival, the original AUC was 0.847 (95%CI: 0.701-0.964); (B) Histogram and fitted curve with AUC values repeated 1000 times for 5-year overall survival, the original AUC was 0.794 (95%CI: 0.714-0.896); (C) Histogram and fitted curve with C-index values repeated 1000 times, the original C-index was 0.895 (95%CI: 0.809-0.952).
